# Evaluation of EELS spectrum imaging data by spectral components and factors from multivariate analysis

**DOI:** 10.1093/jmicro/dfx091

**Published:** 2017-11-09

**Authors:** Siyuan Zhang, Christina Scheu

**Affiliations:** 1 Max-Planck-Institut für Eisenforschung GmbH, Max-Planck-Straße 1, 40237 Düsseldorf, Germany; 2 Materials Analytics, RWTH Aachen University, Kopernikusstraße 10, 52074 Aachen, Germany

**Keywords:** electron energy loss spectroscopy, spectrum imaging, multivariate analysis, non-negative matrix factorization, component-based supervision, oxide interface

## Abstract

Multivariate analysis is a powerful tool to process spectrum imaging datasets of electron energy loss spectroscopy. Most spatial variance of the datasets can be explained by a limited numbers of components. We explore such dimension reduction to facilitate quantitative analyses of spectrum imaging data, supervising the spectral components instead of spectra at individual pixels. In this study, we use non-negative matrix factorization to decompose datasets from Fe_2_O_3_ thin films with different Sn doping profiles on SnO_2_ and Si substrates. Case studies are presented to analyse spectral features including background models, signal integrals, peak positions and widths. Matlab codes are written to guide microscopists to perform these data analyses.

## Teaser

In this paper, we employ the dimension reduction from multivariate analysis to evaluate EELS spectrum imaging data. Signal integration of the Sn-M4,5 edges with delayed onset and the profile of the Fe-L3 white line are quantitatively analysed based on supervision of three spectral components instead of individual pixels.

## Introduction

Since the early days of electron energy loss spectroscopy (EELS), multivariate analysis (MVA) has been introduced to process EELS data [1]. As advancing EELS acquisition routines generate increasingly bigger datasets [2,3], MVA has been widely acknowledged as a powerful tool to process EELS datasets, including spatially resolved spectrum imaging in two dimensions [4–11] and three dimensions [12–14], and sets of angular-resolved [15] or site-specific [16] spectra. With the development in MVA algorithms, principal component analysis (PCA) [17], independent component analysis [18], non-negative matrix factorization (NMF) [19] and geometric extraction methods [20] (such as vertex component analysis and Bayesien linear unmixing [21]) have been applied to treat EELS data. These methods have shown success in noise reduction and have been applied to identify fine structures either around the absorption edge onset or in the valence EELS regime.

MVA algorithms decompose a spectrum imaging dataset into the linear combination of a few spectral components, as expressed by Eq. (1),

(1) $$f_{p}\left(E\right)=\;\underset{i}{\sum }c_{p,i}f_{i}\left(E\right)$$

where *f*_*p*_ and *f*_*i*_ are the EELS spectra as functions of the energy loss *E* for individual pixels *p* and individual spectral components *i*, respectively, and *c*_*p,i*_ are the coefficients for the linear combination. Equation (1) is usually an approximation, neglecting the residual term after the linear combination. At each pixel *p*, *f*_*p*_ is expressed as a linear combination of *i* spectral components *f*_*i*_ with the coefficients *c*_*p,i*_, which is also known as the score matrix. As the number of components *i* is usually much smaller than the number of pixels *p*, *f*_*i*_ is a sparse representation of *f*_*p*_, reducing its dimension by *p*-*i*. Such dimension reduction has enabled a concise representation of spectrum imaging datasets by a number of spectral components with their corresponding coefficients at each pixel for each component.

Thus far, MVA has been widely applied to decompose spectrum imaging datasets [4–14]. However, interpretation on the MVA spectral components remains descriptive and qualitative. It is more efficient to capitalize on the dimension reduction and evaluate the spectral components instead of the spectrum at individual pixels. The computing power will not be saved by such an approach, as the MVA algorithms themselves take more time to perform. On the other hand, the man power to supervise and present the spectrum imaging can greatly benefit from a dimension reduction.

In this article, we demonstrate the component-based supervision to evaluate EELS spectrum imaging data, and bridge the gap between MVA analysis and conventional (pixel-based) EELS quantification routines. Evaluation of quantitative information from EELS spectra requires proper modelling. Model-based quantification, such as EELSModel developed by Verbeeck *et al.* [22], composes an EELS spectrum by element-specific edges and background from the preceding edges, which requires detailed information on the constituents in the sample. It is more common to analyse one absorption edge at a time using assigned energy windows in the vicinity of the edge for background subtraction and signal integration [23]. The selection of energy windows is a necessary supervision for EELS analysis. Enforcing such supervision on individual pixels is a laborious work, prone to bias, and hence rarely done in practice. Making use of the dimension reduction from MVA analysis, supervision on the component level becomes practical, as the linear combination coefficients *c*_*p,i*_ from MVA algorithms are unsupervised and unbiased. We examine two case studies to evaluate the signal of an absorption edge and the chemical shift of a white line feature, respectively, all based on the component-based supervision.

## Data Acquisition

We used thin film hematite (α-Fe_2_O_3_) photoelectrodes as materials systems for the case studies, where Sn dopants are introduced to enhance their photoelectrochemical activity for solar-driven hydrogen production [24]. The distribution of Sn dopants is discussed for three cases, uniformly doped Sn (denoted as Sn: Fe_2_O_3_), partially doped Sn (denoted as Sn/: Fe_2_O_3_), and without Sn dopants (Fe_2_O_3_). Two quantities are of interest from the EELS spectrum imaging datasets, the signal from the Sn-M_4,5_ edges to evaluate the elemental distribution, and the chemical shift of the Fe-L_3_ white line that shows the oxidation state of Fe.

Electron-transparent specimens were prepared by Ar^+^ ion milling and investigated in a FEI Titan Themis microscope operated at 300 kV. Aberration correction of the probe forming lenses enables a scanning probe of 24 mrad convergence semi-angle and ~1 Å probe size. Scanning transmission electron microscopy (STEM) images were collected by the annular bright field (ABF) and the high angle annular dark field (HAADF) detectors that cover the range of 8–16 mrad and 73–352 mrad, respectively. STEM-EELS spectrum imaging was acquired using a Gatan Quantum ERS energy filter in the image-coupled mode with a 35 mrad entrance aperture. Dual EELS collection mode enabled the zero loss peak (ZLP) alignment at individual pixels. Spectra were acquired from relatively thin areas with thicknesses of 0.3–0.5 times the inelastic mean free path. Therefore, the spectra primarily consist of single-scattered inelastic events so that deconvolution with the help of the low loss spectra was not conducted. To minimize specimen damage, charging, and the subsequent drift, moderate dose (0.1 nA probe current and 0.1 s pixel acquisition time) was used to collect spectrum imaging data at a 1 nm × 1 nm pixel size.

## Data Analysis

**Fig.1.**
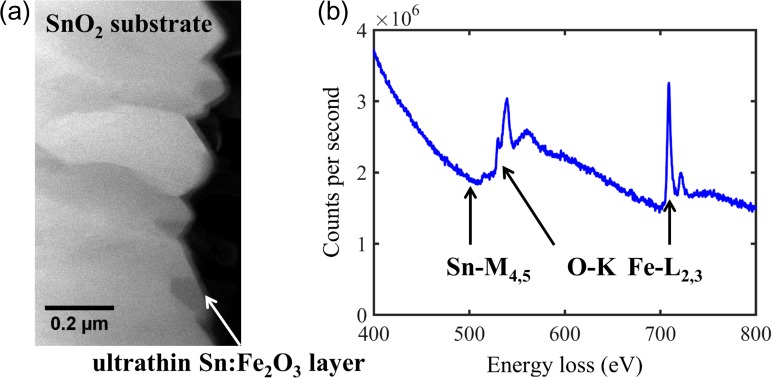
(a) HAADF-STEM micrograph of an ultrathin Sn:Fe_2_O_3_ photoanode on the fluorine doped SnO_2_ substrate. (b) The sum core loss spectrum of an EELS spectrum imaging.

As shown in Fig. 1a, the thin film photoelectrode grown by atomic layer deposition is much thinner than the fluorine doped SnO_2_ substrate. This makes quantification of Sn dopants in the hematite thin films by energy dispersive X-ray spectroscopy very challenging, as nonlocal X-ray excitation (e.g. due to stray electrons and fluorescence artefacts) from SnO_2_ contributes to the signal. On the other hand, EELS only collect forward scattered electrons (up to 35 mrad in this study), so that the signal only contains local inelastic scattering events [23]. The most convenient edges to study are Sn-M_4,5_ edge, which has a delayed edge onset from ~480 eV and overlaps with the O-K edge from ~530 eV, and the Fe-L_2,3_ white lines with onset from ~710 eV, as shown in Fig. 1b.

**Fig. 2.**
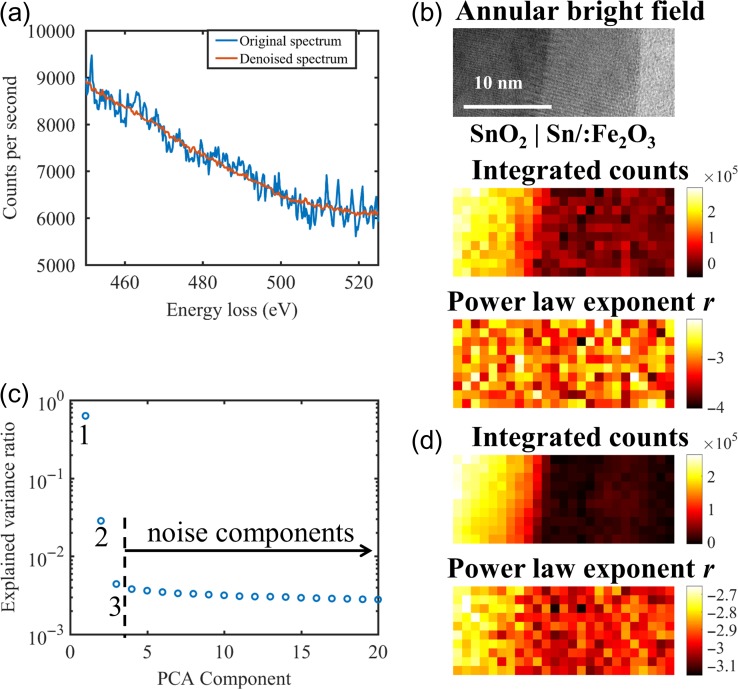
(a) An EELS spectrum from the spectrum imaging dataset before and after the PCA denoise routine, (b) the area for spectrum imaging, the integrated counts of the Sn-M_4,5_ edge, and the power law exponents for background modelling (pixel size is 1 nm), (c) PCA scree plot of the spectrum imaging to identify the threshold level of noise and (d) maps of the integrated counts and the power law exponents as in Fig. 2b after the PCA denoise routine.

First, we show an example on the conventional evaluation (analysed pixel by pixel) of a spectrum imaging across the SnO_2_|Sn/:Fe_2_O_3_ interface shown in Fig. 1a. For simplicity, the same supervision was applied for each pixel here, including the edge integration window (500–525 eV to avoid overlapping with O-K), the background model (power law), and the background fitting window (450–480 eV). Adjusting these parameters for individual pixels is not only exhaustive, but prone to bias. Due to the moderate dose, the spectrum at each pixel is noisy, as shown in Fig. 2a. Consequently, the power law modelling of the background returns noisy parameters, as shown in Fig. 2b, and hence the signal integral is also noisy. There is no finer detail in the signal integral than the higher amount found in SnO_2_ with respect to Sn/:Fe_2_O_3_.

Noise reduction through PCA is a well-established routine in the EELS community, which has been implemented in the Digital Micrograph software as the MSA plug-in [25]. Application of PCA routine to the dataset returns a scree plot, as shown in Fig. 2c, which aligns the spectral components with respect to their explained variance. It is clear that two components stand out explaining the most variance from the dataset, whereas the third component is slightly higher than the following components showing a continuous decay of explained variance from the dataset. In this case, we treat the components starting from the fourth as noise components and set their *c*_*p,i*_ coefficients to 0 to reconstruct the dataset from the first three components, resulting in a denoised spectrum imaging. Figure 2a shows a denoised spectrum at the same pixel to compare with the original one. As shown in Fig. 2d, the power law background fitting on the denoised spectrum imaging returns less noisy parameters and the signal integral is consequently clearer. It is evident from the denoised signal integral that the top half of the Sn/: Fe_2_O_3_ film has more Sn than the bottom half. The different Sn doping level in the Sn/: Fe_2_O_3_ film (top half doped, bottom half nominally undoped) was engineered in the atomic layer deposition process and can only be verified in this example after the PCA denoise routine.

By a sparse representation of an EELS spectrum, MVA analysis not only enables signal identification and noise removal, but also offers the opportunity to supervise on the individual components instead of individual pixels. This can be expressed by the following equation,

(2) $$\underset{E}{\sum }I_{p}\left(E\right)\;=\;\underset{E}{\sum }\underset{i}{\sum }c_{p,i}I_{i}\left(E\right)\;=\;\underset{i}{\sum }c_{p,i}\underset{E}{\sum }I_{i}\left(E\right)$$

where $\;I_{p}\left(E\right)\;=\;f_{p}\left(E\right)-f_{p}^{BG}\left(E\right)$ is the signal of the absorption edge obtained after background subtraction of the EELS spectrum $f_{p}\left(E\right)$ at each pixel, and $I_{i}\left(E\right)\;=\;f_{i}\left(E\right)-f_{i}^{BG}\left(E\right)$ is the signal of the absorption edge for each spectral component. As shown by Eq. (2), summation of the signal counts at individual pixels $I_{p}\left(E\right)$ over an energy range is a linear operation that can be done by linear combinations of such summation of the spectral components $I_{i}\left(E\right)$. As a result, to evaluate the signal counts, supervision on background modelling can benefit from the dimension reduction from all pixels $f_{p}^{BG}\left(E\right)$ to all spectral components $f_{i}^{BG}\left(E\right)$.

Physically, both the EELS signal and background are expected to be positive or 0. Moreover, it is intuitive to decompose the spectrum datasets into non-negative coefficients. The NMF approach decomposes the spectrum imaging into non-negative coefficients and non-negative spectral components, and thereby facilitating the interpretation of individual components. With the development of MVA algorithms, the options for decomposing EELS datasets are ever increasing [19,20]. The decomposed spectral components may all serve as input for further processing. In the following examples, we use the NMF algorithm as described in the scikit learn toolkit [26], which is also used in the open source Hyperspy software [27]. We then focus on the post processing of the MVA spectral components to extract quantitative values for EELS analysis. The following evaluation routines are written in a Matlab package that offers a guided way to process EELS spectrum imaging datasets [28].

**Fig. 3.**
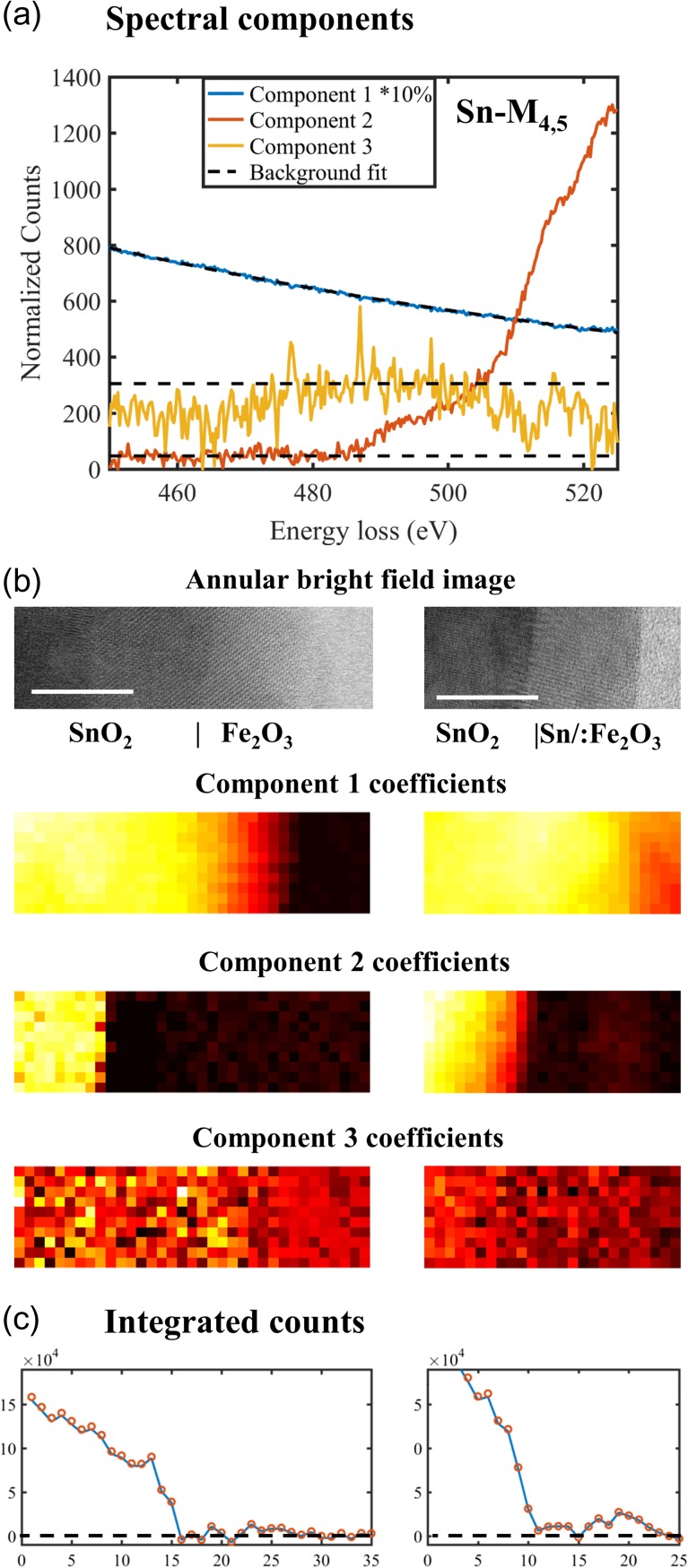
(a) Background fitting as a power law function e^28.14^ × *E*^-3.14^ for component 1, and as constants 47.09 and 305.07 for components 2 and 3, respectively, (b) areas of the spectrum imaging and their respective coefficient maps (scale bars are 10 nm, pixel size is 1 nm), and (c) the signal integration of a line profile derived from Eq. (2) (open circles) as compared with the values from fitting on individual pixels (lines). Dashed lines are eye guides for the baseline (0 integrated counts).

We continue with the aforementioned example to show the evaluation of Sn-M_4,5_ edge counts from component-based analysis and background modelling. In addition to the dataset on the SnO_2_|Sn/:Fe_2_O_3_ interface, another dataset on the SnO_2_|Fe_2_O_3_ interface is included that contains spectra from pure Fe_2_O_3_ to compare with. The combined datasets are decomposed by NMF into spectral components (Fig. 3a) and their corresponding coefficient maps (Fig. 3b). The spectral components are normalized with respect to their corresponding coefficient maps that are set to have an average value of 1. The NMF component 1 has higher coefficients on the sample and follows a power law profile, which reflects the background. Component 2 has higher coefficients where Sn is abundant and reveals the profile of Sn-M_4,5_ edge with a delayed onset. Component 3 has a noisy origin and does not show spectral characteristics from any edges of interest.

Going beyond the qualitative description, background modelling on individual components is required for quantitative analysis. Fitting the background of component 1 is straightforward, as it follows a power law (in the form of $aE^{-r}$) with exponent *r* = 3.14 over the modelled energy range. The signal integration for this component is 0. It is obvious that components 2 and 3 do not follow a power law function within any range of energy loss. Although, linear combination of power law functions has been used to model EELS backgrounds [29], such combinations do not return a power law function. In order to quantify the signal integral of the Sn-M_4,5_ edge, the background of components 2 and 3 is modelled by functions that can be combined linearly, for example, linear functions. As shown in Fig. 3a, the background of component 2 was modelled as a constant before the edge onset, whereas the background of component 3 was also chosen as a constant before the signal integral window to minimize the effect of the preceding feature. The signal integrals of components 2 and 3 are calculated, and those of individual pixels are reconstructed using Eq. (2). The resulting evaluation of signal integral is very close to the evaluation from individual pixels, as shown by the line profile in Fig. 3c. The bottom half of the Sn/:Fe_2_O_3_ film (right spectrum imaging) has the same level of Sn counts as the pure Fe_2_O_3_ film (left spectrum imaging) and the vacuum area. Therefore, the amount of Sn between SnO_2_ and the top half of Sn/:Fe_2_O_3_ is below the detection limit, in accordance with the designed film doping profile. In the top part of the Sn/:Fe_2_O_3_ film (right spectrum imaging), a Sn signal is detected. In this example, component-based supervision reproduces the quantification from the conventional (pixel-based) routine, while the supervision on the background model for three components replaces the supervision on 650 pixels.

Despite having only three spectral components, supervision on the background subtraction is far from straightforward, as there is no definite function to model. In the example above, the background is modelled by the linear combination of a power law function and two constants, as shown in Fig. 3a. The power law exponent *r* = 3.14 of component 1 is close to the maximum exponent among individual pixels, as shown in Fig. 2d. Adding constants to such a background effectively reduces the power law exponent. In order to quantify the difference between power law functions, we make use of the fact that the multiplication of two power law functions returns a power law function. Instead of decomposing the spectrum imaging dataset into spectral components, the spectrum imaging can be factorized into spectral factors,

(3) $$f_{p}\left(E\right)\;=\;\prod_{i}f_{i}{\left(E\right)}^{c_{p,i}},\;\text{or}\;\ln f_{p}\left(E\right)\;=\;\underset{i}{\sum }c_{p,i}\ln f_{i}\left(E\right)$$

In order to realize factorization by means of linear MVA algorithms, a logarithmic operation needs to be taken, as formulated in Eq. (3). In comparison with Eq. (1), $f_{p}\left(E\right)$ and $f_{i}\left(E\right)$ are replaced by their logarithmics. The practical implementation includes taking the logarithmic of EELS spectrum $\ln f_{p}\left(E\right)$ as input for MVA algorithms, and taking the exponents of the output $\ln f_{i}\left(E\right)$ as spectral factors.

**Fig. 4.**
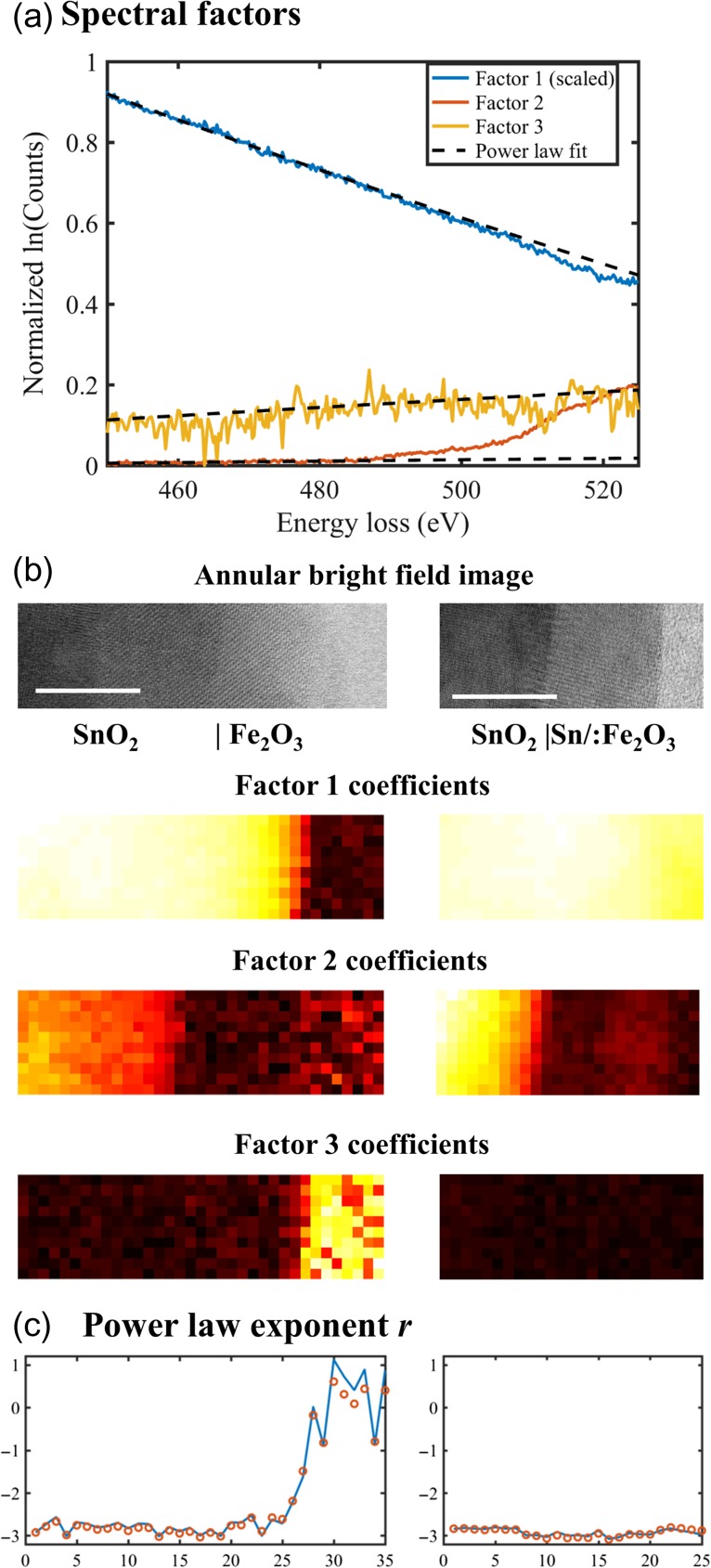
(a) Background fitting parameters for the three spectral factors (factor 1 scaled down by ln(2000) = 7.6): (*a*_1_, *r*_1_) = (26.30, 2.91), (*a*_2_, *r*_2_) = (−2.83, −0.48), (*a*_3_, *r*_3_) = (−0.50, −0.08), (b) areas of the spectrum imaging and their respective coefficient maps (scale bars are 10 nm, pixel size is 1 nm), (c) the power law exponent of a line profile reconstructed from Eq. (4) (open circles) as compared with the values from fitting on individual pixels (lines).

As an example, the EELS spectrum imaging shown in Fig. 3 was taken the logarithmic operation and underwent NMF decomposition to factorize into spectral factors (Fig. 4a) and their corresponding coefficient maps (Fig. 4b). As shown in Fig. 4a, background modelling on the spectral factors $\ln f_{i}\left(E\right)$ becomes more straightforward using a linear fit against ln (*E*) before the edge onset. In order to reconstruct the power law background of individual pixels, the form of the power law function is put into Eq. (3), which is then rearranged into Eq. (4),

(4) $$\begin{array}{c} \ln(a_{p}E^{-r_{p}})=\underset{i}{\sum }c_{p,i}\ln(a_{i}E^{-r_{i}}),\text{where}\;\ln a_{p}\\ \;=\;\underset{i}{\sum }c_{p,i}\ln a_{i},\;\text{and}\;r_{p}=\underset{i}{\sum }c_{p,i}r_{i} \end{array}$$

where *a*_*i*_ and *r*_*i*_ are the prefactor and the exponent of the power law function for each spectral factor, respectively, and *a*_*p*_ and *r*_*p*_ are the power law parameters at each pixel. In this example, the component-based supervision allows for a selection of different windows for the power law fitting of the background. As shown in Fig. 4a, spectral factors $\ln f_{i}\left(E\right)$ for *i* = 1, 2 are linear with respect to ln(*E*) in the window between 450 and 480 eV, before the Sn-M_4,5_ edge onset. Factor 3, on the other hand, is not linear in that range, as a dip is observed around 465 eV, and the fitting window is chosen between 475 and 495 eV. From the coefficient map in Fig. 4b, it is shown that factor 3 mainly contributes to the area beyond the thin film (the glue line and vacuum) of the left spectrum imaging, which does not relate to the Sn-M_4,5_ edge. The power law exponents for each pixel are then reconstructed from the three factors and their coefficient maps according to Eq. (4), as shown in Fig. 4c. A comparison there with a pixel by pixel power law fitting using a fixed window shows a good agreement between the two approaches. Fitting the background from the spectral factors offers a more straightforward supervision by offering the same function of power law (or a linear function in the logarithmic form, see Fig. 4a) and the flexibility on the fitting window.

**Fig. 5.**
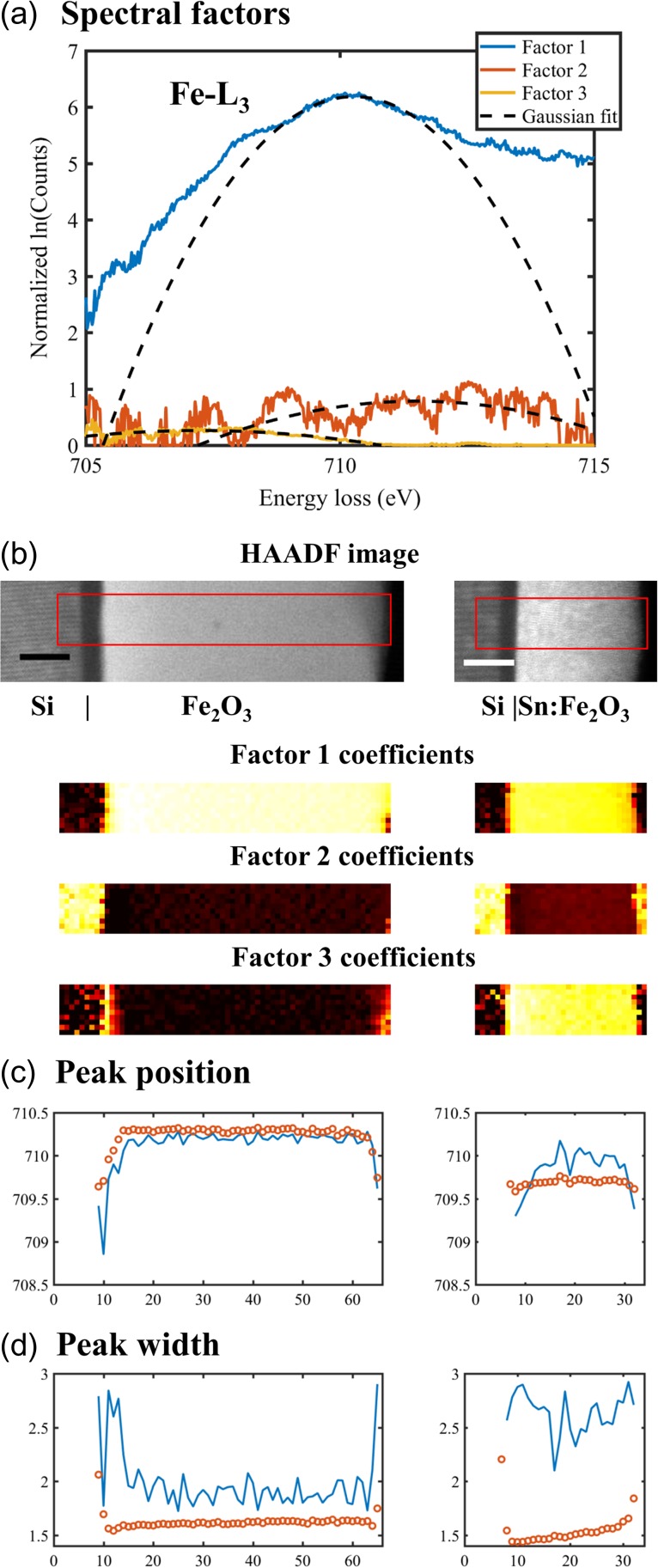
(a) Gaussian fitting parameters for the 3 factors (*a*_1_, *μ*_1_, *σ*_1_) = (6.187, 710.274, 1.403), (*a*_2_, *μ*_2_, *σ*_2_) = (0.783, 711.519, 3.447), (*a*_3_, *μ*_3_, *σ*_3_) = (0.262, 707.236, 4.935), (b) areas of the spectrum imaging and their respective coefficient maps (scale bars are 10 nm, pixel size is 1 nm); (c) the centre and (d) the width of a line profile derived from Eq. (5) (open circles) are compared with the values from Gaussian fitting of individual pixels (lines).

Besides modelling the power law background, the logarithmic formulation Eq. (3) can be useful to study other mathematic functions that can be factorized. For example, the Gaussian shape function can be modelled using the following expression,

(5) $$\begin{array}{c} \ln(a_{p}e^{-\frac{{\left(E-\mu_{p}\right)}^{2}}{2\sigma_{p}^{2}}})=\underset{i}{\sum }c_{p,i}\ln(a_{i}e^{-\frac{{\left(E-\mu_{i}\right)}^{2}}{2{\sigma_{i}}^{2}}}),\;\text{where}\\ \;\sigma_{p}^{-2}=\underset{i}{\sum }c_{p,i}\sigma_{i}^{-2},\;\text{and}\;\mu_{p}=\frac{\sum_{i}c_{p,i}\mu_{i}\sigma_{i}^{-2}}{\sum_{i}c_{p,i}\sigma_{i}^{-2}} \end{array}$$

where *a*_*i*_, *μ*_*i*_ and *σ*_*i*_ are the height, the centre, and the width of the Gaussian shape function for each spectral factor, respectively, and *a*_*p*_, *μ*_*p*_ and *σ*_*p*_ are the Gaussian shape parameters at each pixel, which can be reconstructed using Eq. (5). We use the Fe-L_3_ peak as a case study for Gaussian fitting to determine the chemical shift. As shown in Fig. 5b, two datasets are combined together, representing a scan across a Si|Fe_2_O_3_ interface for the left dataset and a scan across a Si|Sn:Fe_2_O_3_ interface for the right. The background of the Fe-L_3_ peak was modelled by supervising individual spectral factors and reconstructing using Eq. (4), as introduced in the preceding example. Then, the Fe-L_3_ peak signal is subjected to the NMF decomposition in the logarithmic formulation Eq. (3) into the spectral factors (Fig. 5a) and their corresponding coefficient maps (Fig. 5b).

A quadratic fitting on each spectral factor is performed to model *a*_*i*_, *μ*_*i*_ and *σ*_*i*_ of the Gaussian peak. In comparison with the centre of factor 1, factor 2 represents a shift to the right (*μ*_2_ > *μ*_1_), whereas factor 3 represents a shift to the left (*μ*_3_ < *μ*_1_). It is shown in Fig. 5d that factor 1 comes from undoped and doped Fe_2_O_3_ of both spectrum imaging, factor 2 mainly from areas without Fe_2_O_3_, and factor 3 from the homogeneously doped Sn:Fe_2_O_3_ film (right spectrum imaging) as well as the interface and surface of undoped Fe_2_O_3_ film (left spectrum imaging). A chemical shift of the Fe-L_3_ edge correlates to the bonding environment and especially the oxidation state of Fe atoms. As hematite (undoped Fe_2_O_3_) only consists of Fe^3+^, a chemical shift to lower energy loss indicates a lower oxidation state of Fe, for example, reduction to Fe^2+^[30].

A reconstruction of the Gaussian peak parameters using Eq. (5) then returns a left shift of the peak at the pixels where factor 3 has large coefficients, as shown by a line profile in Fig. 5c. The quantified Gaussian parameters are compared with those fitted for individual pixels in Fig. 5c and 5d, where a discrepancy is evident. This is because modelling the spectral factors (Fig. 5a) as Gaussian functions has a big discrepancy. For example, factor 1 has a noticeable deviation from a Gaussian shape, showing a broader profile. As a result, the width derived from spectral factors is lower than the width modelled at individual pixels, as shown in Fig. 5d. Moreover, factor 2 has several peak features, whereas the modelled peak only takes their enveloped shape into account. Despite the conventional modelling of the white line features by Gaussian peaks, a spectrum imaging may be impossible to be factorized into Gaussian peaks. Indeed, many more types of functions have been applied to model a white line feature to model the chemical shift [30].

In order to propose a universal way to model the peak position, we return to the linear formulation Eq. (1). Instead of doing peak fitting, we approximate the centre and the width of the peak profiles by their statistical mean and variance, as shown by the following expressions,

(6) $$\begin{array}{c} \begin{array}{cc} \mu_{p} & =\;\frac{\sum_{E}EI_{p}\left(E\right)}{\sum_{E}I_{p}\left(E\right)}\\ & =\;\frac{\sum_{E}E\sum_{i}c_{p,i}I_{i}\left(E\right)}{\sum_{E}\sum_{i}c_{p,i}I_{i}\left(E\right)}=\;\frac{\sum_{i}c_{p,i}\sum_{E}EI_{i}\left(E\right)}{\sum_{i}c_{p,i}\sum_{E}I_{i}\left(E\right)} \end{array} \end{array}$$

(7) $$\begin{array}{c} \begin{array}{cc} \sigma_{p}^{2} & =\;\frac{\sum_{E}{\left(E-\mu_{p}\right)}^{2}I_{p}\left(E\right)}{\sum_{E}I_{p}\left(E\right)}=\frac{\sum_{E}E^{2}I_{p}\left(E\right)}{\sum_{E}I_{p}\left(E\right)}-{\left(\frac{\sum_{E}EI_{p}\left(E\right)}{\sum_{E}I_{p}\left(E\right)}\right)}^{2}\\ & =\;\frac{\sum_{i}c_{p,i}\sum_{E}E^{2}I_{i}\left(E\right)}{\sum_{i}c_{p,i}\sum_{E}I_{i}\left(E\right)}-\frac{{\left(\sum_{i}c_{p,i}\sum_{E}EI_{i}\left(E\right)\right)}^{2}}{{\left(\sum_{i}c_{p,i}\sum_{E}I_{i}\left(E\right)\right)}^{2}} \end{array} \end{array}$$

where $\mu_{p}$ and $\sigma_{p}$ are the statistical mean and variance at each pixel, and the other variables are defined in Eq. (2). Similar to Eq. (2), individual terms in the numerators and denominators of Eqs. (6) and (7) can be expressed as linear combination of quantities from the spectral components, including their first and second moments. Unlike the previous example (Fig. 5) where each spectral factor is fit to a Gaussian shape, the spectral components discussed here do not need to have a Gaussian shape to derive their moments for the reconstruction of the peak shape parameters at each pixel using Eqs. (6) and (7).

**Fig. 6.**
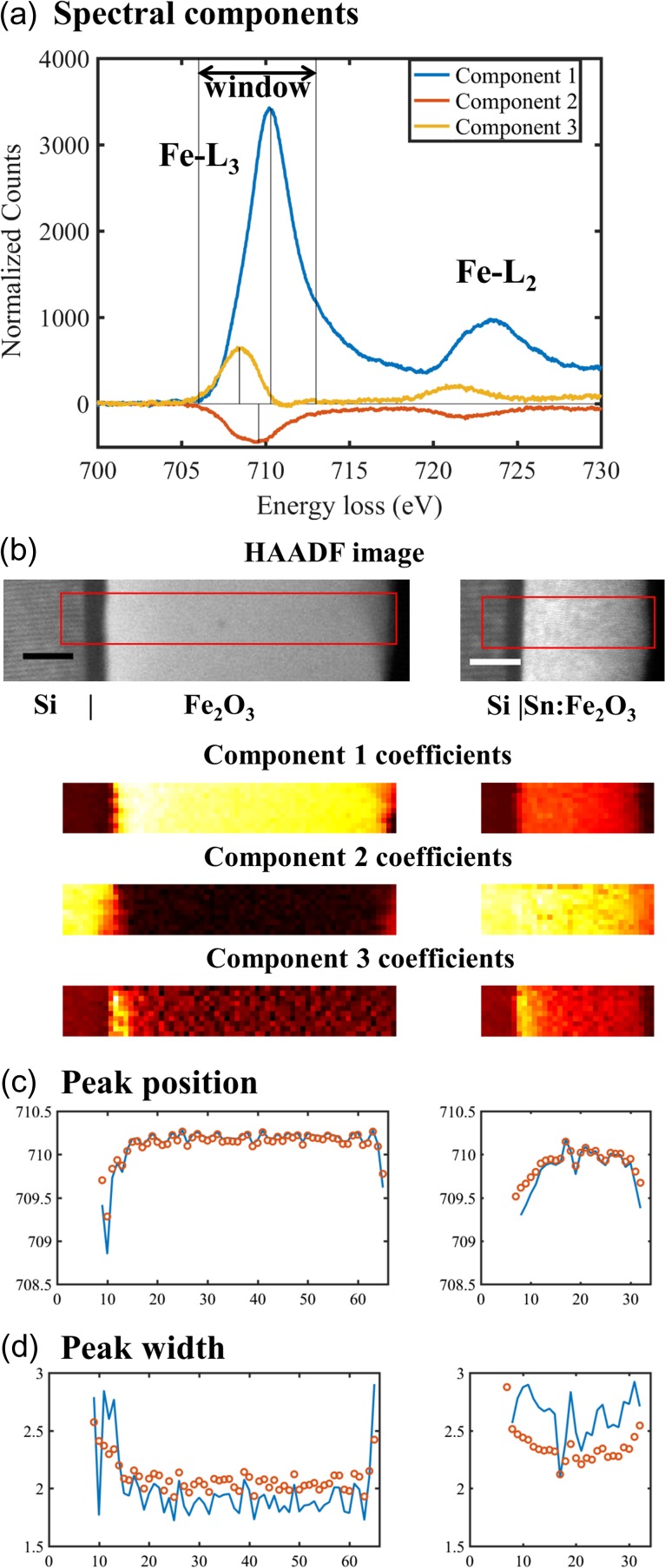
(a) Spectra of the three components after background subtraction with supervised window selection, (b) areas of the spectrum imaging and their respective coefficient maps (scale bars are 10 nm, pixel size is 1 nm); line profiles of the Fe-L_3_ (c) peak position and (d) peak width derived from individual components (open circles) are compared with the values from Gaussian fittings of individual pixels (lines).

As an example, the Fe-L_3_ peak signal from the preceding example underwent NMF decomposition in the linear formulation Eq. (1) to compute the spectral components (Fig. 6a) and their corresponding coefficient maps (Fig. 6b). The background of each spectral component is modelled by power law and linear fitting introduced in the example of Fig. 3. Figure 6a displays the spectral components after background subtraction, where negative values may show up (e.g. component 2). The supervision on each spectral component is necessary to find an energy window so that the resulting centre of intensity coincides with their respective peak positions, as shown in Fig. 6a. By using this approach, there is a much improved numerical agreement on the peak position with the Gaussian analysis at individual pixels, as shown in Fig. 6c. Moreover, as shown in Fig. 6d, the peak width reconstructed from the variance of individual components has the same trend as the Gaussian width determined from individual pixels. Component-based supervision on the window selection can be a reliable way to reconstruct the position (chemical shift) and the width of a peak feature and replace the supervision and curve fitting for individual pixels.

## Conclusion

In summary, we have employed MVA analyses to reduce the dimension of EELS spectrum imaging datasets and thereby facilitating the supervision for further evaluation. This has been employed to model the integral counts as well as the centre and width of peak features. Using the logarithmic formulation introduced to factorize the dataset, parameters of power law background can be reconstructed, and deviation from a Gaussian peak is visualized. These utilities are implemented in a Matlab package. More utilities to model and analyse EELS spectrum imaging datasets can benefit from component-based supervision facilitated by MVA analysis.
